# Move for Change Part II: a European survey evaluating the impact of the EPDA Charter for people with Parkinson's disease

**DOI:** 10.1111/j.1468-1331.2012.03876.x

**Published:** 2012-10-03

**Authors:** F Stocchi, B R Bloem

**Affiliations:** a Department of Neurology, Institute for Research and Medical Care, IRCCS San RaffaeleRome, Italy; b Department of Neurology, Donders Institute for Brain, Cognition and Behaviour, Radboud University Nijmegen Medical CentreNijmegen, the Netherlands

**Keywords:** Charter, European Parkinson's Disease Association, European survey, funding, Parkinson's disease, quality of care, support services

## Abstract

**Background and purpose:**

The Move for Change campaign is a three-part series of pan-European surveys designed by the European Parkinson's Disease Association (EPDA) to assess the impact that the EPDA Charter for People with Parkinson's disease (PD) has had since its launch in 1997. Here, we report results from the second survey, focusing on the third right of the Charter; that is, ‘all patients have the right to have access to support services’. Although the level of evidence for different support services varies, it is important to ensure that patients can access services with clinically proven benefits.

**Methods:**

This survey comprised nine questions administered online via the EPDA and PD organization Web sites. Accessibility of support services was defined as ‘*services/medication/multidisciplinary healthcare professionals, etc. being available and on hand to patients when required*’.

**Results:**

Neurologists and general practitioners (GPs) received highest accessibility results (90.0 and 87.0% of respondents, respectively), with moderate results for physiotherapists (68.0%) and PD organizations (72.0%) and lower results for PD specialist nurses (26.0%), occupational therapists (23.0%), and counselors (27.0%). Support provided by neurologists and PD specialists was considered to be ‘very helpful’ by 59.0 and 55.7%, respectively, whilst only 31.8% of respondents gave such favorable ratings to GPs. Funding of services was variable across Europe.

**Conclusions:**

These data demonstrate the challenges faced by PD patients in accessing the adequate care and support required throughout the course of their disease. These findings can assist healthcare professionals and policymakers in improving access to support services for patients and their families across Europe.

## Introduction

Parkinson's disease (PD) affects approximately 1.2 million people across Europe [1]. The neurodegenerative nature of the disease can lead to a greatly diminished quality of life (QoL) [2–4]. This is reflected in the socioeconomic burden of PD, estimated to be €13.9 billion across Europe [5,6]; this burden is expected to worsen as aging populations face increased risk of PD [7,8]. Early and effective management of chronic conditions may significantly reduce treatment costs, for example, potentially delaying the institutionalization of a patient, an event associated with a 500% cost increase, due to disease-modifying effects [9,10].

The European Parkinson's Disease Association (EPDA) has campaigned for 20 years to improve the standards of care for PD patients across Europe. In conjunction with the World Health Organization, it launched the *Charter for People with Parkinson's Disease* in 1997 aimed at raising the profile of PD and enhancing the public's awareness of the disease [11]. Developed with input from patients and a Medical Advisory Board (including European neurologists), the Charter demands minimum standards of care for PD patients and states that all patients have the right to: be referred to a doctor with a special interest in PD; receive an accurate diagnosis; have access to support services; receive continuous care; and take part in managing their illness [11]. To assess the impact the Charter has had across Europe, the Move for Change (MfC) campaign was launched in 2010, a three-part series of pan-European surveys to identify the aspects that have been achieved and where more assistance is required [12]. The results from Part I of the survey summarized data from over 2000 completed questionnaires received from 35 European countries [13]; this manuscript indicated that only 11.9% of respondents had received their initial diagnosis from a PD specialist, and 43.8% of the respondents had not received a consultation from a PD specialist in the 2 years following diagnosis. Furthermore, almost half the patients were dissatisfied with the manner in which the eventual diagnosis was delivered.

To coincide with the publication of the MfC Part I results, a multidisciplinary team of healthcare professionals (HCPs), Parkinson's specialists, patients, and their carers worked with the EPDA to develop *The European Parkinson's Disease Standards of Care Consensus Statement Volume I* [14]. This collaboration aimed to demonstrate the standards of care that PD patients should receive, and the document will be used with the MfC survey results to lobby for standardization of Parkinson's care across Europe; the Consensus Statement was presented to Members of the European Parliament in November 2011.

This article presents the findings of Part II of the MfC campaign. The European survey was carried out in 2011 and concentrates on the third point of the Charter: that is, ‘all patients have the right to have access to support services’. Effective management of Parkinson's requires a holistic approach involving a multidisciplinary team [14,15], including physiotherapists, speech and language therapists, and occupational therapists [15,16]. This second survey asked participants to rate a variety of services in relation to their accessibility (operationally defined as *‘services/medication/multidisciplinary HCPs, etc. being available and on hand to patients when required*’) and level of helpfulness.

## Methods

The MfC survey Part II was launched online on 12 April 2011 in conjunction with the European Parkinson's Action Day and ran until 31 October 2011. Promotion of this survey included use of translated Web site banners; advertisements in quarterly Member Organizations' (MOs') national journals and external journals; e-mails from MOs to their members; and promotion by the EPDA via the EPDA Update, publications, and social media (Twitter and Facebook). It is difficult to determine exactly how many patients were aware of the survey as this was reliant on promotion by the MOs.

The survey complied with the Code of Conduct for pharmaceutical market research of the European Pharmaceutical Market Research Association, and no adverse events were reported. Approval from the Clinical Research Ethics Committee or Independent Review Board was not required because drug therapy was not addressed. The methods used for Part II of this survey have been previously published [13]; however, differences from the Part I survey are highlighted below.

### The survey

The survey comprised nine questions covering: patient demographics; identified areas where additional support is required; the level of access to support services; and the level of assistance provided to access these services. Accessibility was operationally defined as ‘*services/medication/multidisciplinary HCPs, etc. being available and on hand to patients when required*’, and the survey covered the perceived effectiveness and frequency of utilization of the services patients indicated were accessible. Translated into 24 languages by the local associations, the survey was therefore completed in each participant's local language. *The complete, original questionnaire and wording is included as an online supplement for this manuscript*.

## Results

### Assimilation of questionnaire information

A total of 1786 forms were received from patients in 32 countries, and 1752 (98.1%) were analyzed. Thirty-four questionnaires were rejected because the majority of questions were left unanswered. Although results from countries with <9 respondents (i.e., ≤0.5% of the total survey sample) were included in regional- and European-level analyses, data from these countries were not analyzed at an individual country level.

### Demographics

Of the 1752 questionnaires analyzed, 53.0% were from men; the most common age-group was 60–69 years (40.0%); and the ages of respondents ranged from <30 years (0.1%) to ≥ 80 years (4.0%). With regard to year of diagnosis, 1.0% of patients were diagnosed before 1986, whilst 51.0% were diagnosed from 2006 to 2011. Table 1 shows the regional distribution and demographic data for respondents.

**Table 1:** Geographic region distribution of respondents

	Mean gender^a^					
					
Global region	Male (%)	Female (%)	Mean age (years)	Mean years since diagnosis (years)	Forms analyzed (*N*)	Percentage of total forms analyzed (%)
Eastern Europe	47.0	52.0	63.4	8.2	238	13.6
Bulgaria^b^, Czech Republic, Hungary, Poland, Romania^b^, Slovakia^b^, Ukraine						
Northern Europe	56.0	43.0	64.3	6.9	821	46.9
Denmark, Finland, Ireland, Lithuania, Norway, Sweden, UK						
Southern Europe	51.0	49.0	62.6	8.6	263	15.0
Croatia^b^, Cyprus^b^, Greece, Israel, Italy, Malta^b^, Portugal^b^, Slovenia, Spain, Turkey^b^						
Western Europe	53.0	47.0	64.0	7.3	419	23.9
Austria, Belgium, France, Germany^b^, Luxembourg, Monaco^b^, the Netherlands, Switzerland						
Not stated	100.0	0.0	63.6	7.5	11	0.6
Total^a^	53.0	46.0	63.8	7.4	1752	100.0

a One per cent of respondents did not state gender.

b Included in regional analysis; national sample too small to analyze individually.

### Where support is needed

**Figure 1:**
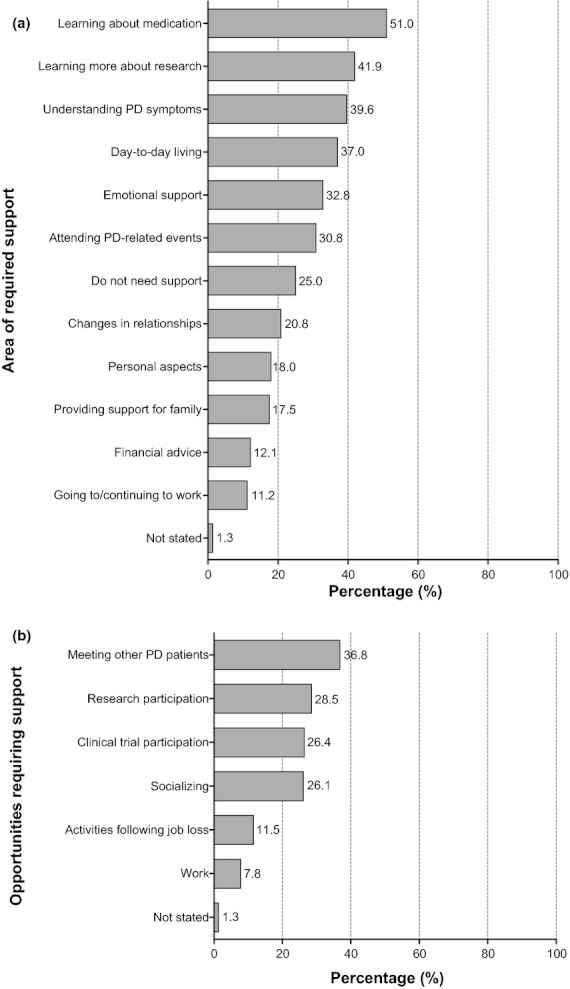
Percentage of total respondents indicating the areas where additional support would be beneficial, listed per support area: (a) areas patients require additional support with (*n* = 1752); (b) opportunities patients require support in finding (*n* = 1752).

The areas where patients have indicated additional support would be beneficial are shown in Fig. 1a; only 25.0% stated that they did not require support, whilst learning about medication was selected by the majority (51.0%). The areas that received the least responses were financial advice (12.1%) and going to/continuing to work (11.2%).

Patients were also asked to indicate the areas where they would benefit from assistance in finding opportunities (Fig. 1b); meeting other PD patients was the most important (36.8%), whilst opportunities related to work were indicated to be the least important (7.8%).

### Access to support

**Figure 2:**
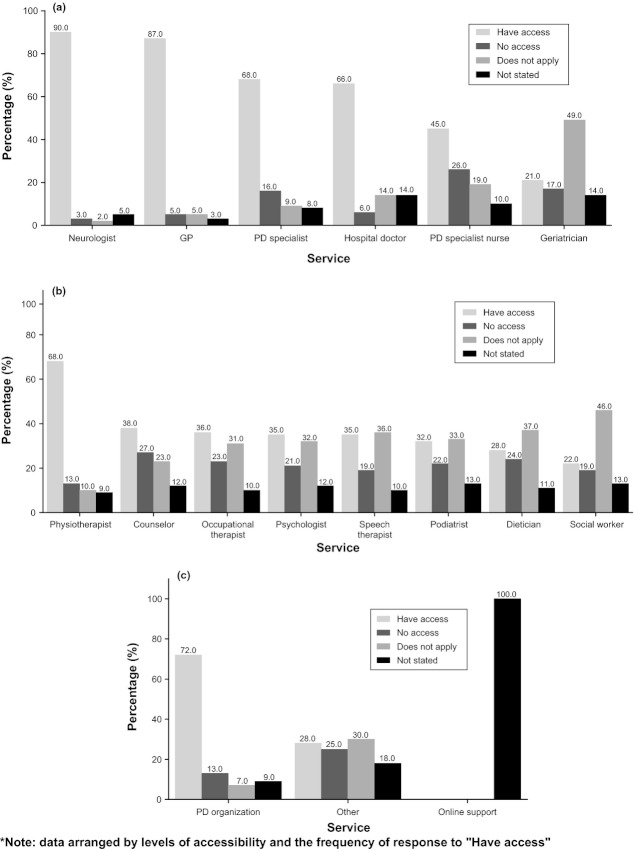
Percentage of total respondents indicating their accessibility to support services, listed and categorized by service: (a) clinical services (*n* = 1752); (b) allied health services (*n* = 1752); (c) other services (*n* = 1752).

Figure 2a shows that the highest accessibility level was reported for neurologists; 90.0% of the respondents indicated they had full access to this service, whilst only 3.0% reported a lack of access. General practitioners (GPs) also received a high rating of accessibility, 87.0%. Access to a PD specialist scored lower (68.0%), despite the EPDA Charter stipulations, and PD specialist nurses were only considered to be accessible by 45.0% of patients, whilst 26.0% indicated a lack of access, and 19.0% indicated that the service was not applicable to them.

In terms of allied health services (Fig. 2b), 68.0% of patients considered physiotherapists to be the most accessible. Occupational, and speech and language therapists were also rated quite highly for accessibility (36.0% and 35.0%, respectively). Finally, PD organizations were rated as accessible by 72.0% of patients, indicating that their support is the most accessible of the allied health support services (Fig. 2c).

### Help with access

**Figure 3:**
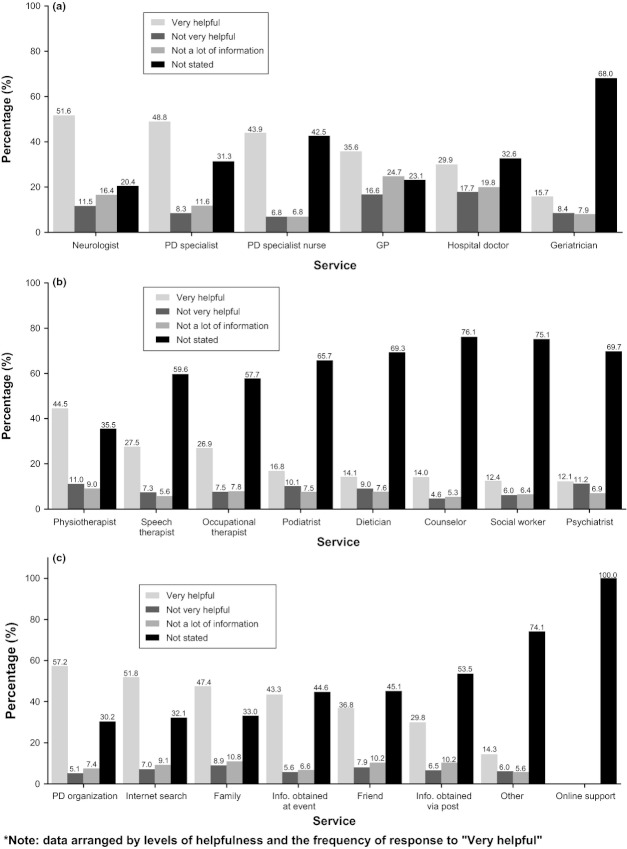
Percentage of total respondents indicating the helpfulness of services to increase patient access to additional support services, listed and categorized by service: (a) clinical services (*n* = 1752); (b) allied health services (*n* = 1752); (c) other services (*n* = 1752).

Patients were asked to identify the services that are the most helpful in enabling them to gain access to additional support services. Neurologists were rated as the most helpful amongst the clinicians, receiving ‘very helpful’ ratings from 51.6% of patients (Fig. 3a). Interestingly, PD specialists were rated as ‘very helpful’ by 48.8% of patients despite being considered to be inaccessible by 16.0% of the survey respondents. Although GPs were accessible to a large proportion of patients, they were thought to ‘not have enough information’ to be of adequate assistance by 24.7% of respondents. PD specialist nurses received mixed ratings of assistance level, which may relate to the varying levels of accessibility to this service across the participating countries; however, it was considered to be a ‘very helpful’ resource by 43.9% of respondents.

The majority of allied health services received ‘not stated’ ratings from 35.5% to 76.1% of participants, suggesting that these services may not apply to a lot of patients (Fig. 3b); however, physiotherapy was indicated as the most helpful service by 44.5% of patients. Figure 3c shows that patients considered PD organizations, the Internet and family to be ‘very helpful’ in assisting in gaining support from other services (57.2%, 51.8%, and 47.4%, respectively).

### Helpfulness of support

**Figure 4:**
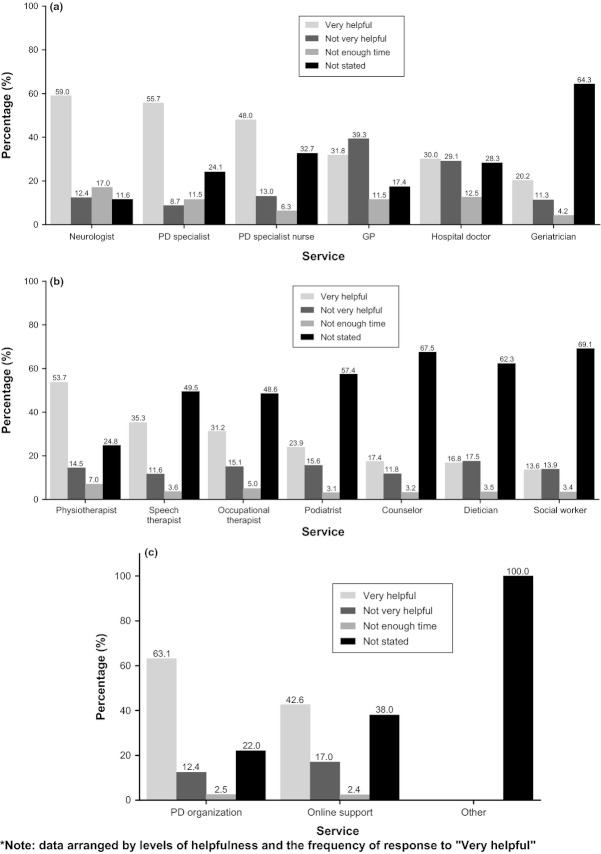
Percentage of total respondents indicating the helpfulness of the support provided by various services, listed and categorized by service: (a) clinical services (*n* = 1752); (b) allied health services (*n* = 1752); (c) other services (*n* = 1752).

Patients were asked to indicate how helpful each of the support services was in relation to patient care; amongst clinicians, neurologists were identified as the most helpful (59.0%; Fig. 4a). Although 55.7% of the total patient cohort rated PD specialists favorably, 24.1% did not rate the service at all; this is twice the proportion who did not rate neurologists (11.6%), suggesting that patients are not accessing PD specialists as often as they should. Patients across Europe had mixed opinions about GPs; although one of the most accessible services, GPs were rated as ‘not very helpful’ by slightly more patients (39.3%) than those who rated them as ‘very helpful’ (31.8%). In addition, the respondents indicated that all of the clinician services ‘did not have enough time’ for them; this includes neurologists (17.0%), the most accessible service.

Physiotherapy was viewed as ‘very helpful’ by 53.7% of patients, confirming that this is the most accessible and helpful allied health service (Fig. 4b). The majority of allied health services were not rated by the patients, suggesting a variable level of accessibility across Europe. Finally, patient organizations were consistently rated favorably across Europe; in several countries, this service was rated as ‘very helpful’ by 63.1% of patients overall (Fig. 4c).

### Funding of support

As Table 2 shows, funding for support services was highly variable across Europe; the full analysis report shows these data at a country level and is available on the EPDA Web site (http://www.epda.eu.com/en/projects/move-for-change/part-2/). The majority of support services in the UK were funded by the government, whilst services in Switzerland were mostly funded by private insurers. Conversely, the funding of treatment by various clinicians in Greece involved a large contribution from the patients themselves.

**Table 2:** Percentage of total respondents indicating how the accessible support services are funded, listed per service

	Funded by					
	
Service	The government (%)	Private insurers (%)	The Parkinson's disease (PD) patient (%)	PD organization (%)	Another organization (%)	Not stated (%)
General practitioner	47.2	15.8	15.9	2.3	2.2	16.6
Hospital doctor	49.0	12.7	11.7	1.0	1.9	23.8
Neurologist	44.4	16.7	19.0	0.7	2.3	16.9
PD specialist	38.7	12.6	16.7	1.7	2.3	27.9
Geriatrician	32.6	5.4	5.7	1.0	1.6	53.7
PD nurse	34.9	13.1	9.6	2.6	2.3	37.5
Physiotherapist	37.9	14.5	17.5	2.0	2.6	25.6
Occupational therapist	30.2	8.8	8.6	2.1	2.7	47.6
Speech therapist	30.2	9.5	8.5	2.4	2.2	47.1
Dietician	24.2	4.9	9.9	2.4	1.5	57.2
Podiatrist	15.3	5.9	30.0	0.8	1.7	46.3
Counselor	13.0	2.8	11.0	5.0	1.4	66.8
Other	7.1	3.0	15.8	5.9	1.3	66.9

Overall in Europe, governmental funding accounted for the majority of clinician costs: GP, 47.2%; neurologist, 44.4%; and PD specialist, 38.7%. The remaining costs were funded either through private insurance or by the patient, although small contributions from patient organizations were observed occasionally. Allied health services were mostly funded by the patients themselves, with podiatry being the most commonly used service funded this way (30.0%).

## Discussion

The MfC survey is the largest of its kind in Europe, devised to identify the areas of PD care which do not meet the standards specified in the EPDA Charter and current clinical guidelines [11,17–21]. Part II of the survey focuses on the accessibility of additional support services, and these data show considerable variation across Europe. Due to the online format of our survey, it was not possible to calculate an accurate response rate in relation to the population of each participating European country; however, consistent findings were seen across the countries, such as the high accessibility to neurologists and the poor accessibility to a number of allied health services.

The survey population is representative of the general European PD population; the average age of participants was 63.8 years, and the mean disease duration was 7.4 years. These data are comparable to both the Part I survey (62.2 years of age, 8.3-year mean disease duration) [13] and a study carried out in 802 PD patients from Spain and Holland, which showed an average age within two PD patient cohorts of 60.8 and 66.2 years, with mean disease duration of 9.9 and 7.7 years, respectively [22].

The combined motor and non-motor symptoms of PD require a multidisciplinary approach to ensure adequate treatment [23], and the EPDA Charter stipulates that access to additional support services should be available for all patients. Although the level of evidence for different support services varies, it is important to ensure that patients can access services with clinically proven benefits. The results from this survey have highlighted that access to allied health services is limited, particularly to occupational therapy (OT), with only 36.0% of respondents indicating access to this service. However, only 11.2% indicated that they require further support with going to/continuing to work; it is possible that the low percentage of work-related responses could demonstrate a lack of understanding from the PD patients. A Finnish study in PD patients <65 years [24] shows that only 16% of 937 PD patients were still working; however, of these, only 18% who were in full-time employment felt that PD had reduced their working capacity. These authors suggested that occupational health services need an increased knowledge of PD and its symptoms. Evidence to support the merits of OT when used alone is still limited (class III evidence at best), but it has been suggested to help improve the QoL of PD patients when used as part of a multidisciplinary approach [25–28]. Limited access to speech therapists was also noted, even though the benefits of speech therapy in improving swallowing motility disorders and vocal function are now supported by class II evidence [29–31]. Access to counselors was limited, but despite their widely felt benefits, the effectiveness of counseling has not been formally studied. Depression is a common symptom of PD [32], and appropriate holistic treatment could improve this [33,34], but requires formal study.

This survey investigated the availability of clinicians at varying levels of specialty; the results suggest that PD specialists remain less accessible than general neurologists and GPs, which correlates with the data from the Part I manuscript where only 11.9% of respondents received their diagnosis from a PD specialist [13]. Retrospective observational studies suggest that clinical outcomes and survival rates in PD are improved with specialist care [35,36]. The accessibility of PD specialists (68.0%) and general neurologists (90.0%) observed here suggests an improvement over the levels observed in earlier studies, for example, only 58% of Medicare PD patients in the United States had seen a neurologist between 2002 and 2005 [36], whilst 10 PD patients were identified from 1780 people visited in Bolivia, none of whom had received a neurological examination or specialist treatment in advance of the study [37].

The availability of specialist PD nurses varied considerably across Europe, with the lowest levels of access reported in Eastern Europe; only 8% of patients indicated access to a PD specialist nurse, whilst 55% advised that the service was inaccessible. Based on everyday clinical experience, many feel that the use of specialist nurses should become common in the treatment for PD, but the supporting evidence is still limited. Some studies suggest that PD nurses can improve the well-being of patients without increasing healthcare costs [38] by assisting in several patient-centered tasks such as education, symptom management, and medication support [39]. However, a later systematic review has suggested that the cost-effectiveness of nursing care for PD remains inconclusive [40]. The costs and clinical effectiveness of a specialist multidisciplinary approach in PD are currently being investigated within the Specialist Parkinson's Integrated Rehabilitation Team Trial [41] and a large trial in the Netherlands [42]. With regard to physiotherapist involvement specifically, 68.0% of respondents indicated they had access to this beneficial service; a cluster-randomized trial has shown that professional networks of physiotherapists specialized in PD improve the quality of care whilst reducing healthcare costs [43].

GPs were one of the most accessible clinical services to PD patients; however, they also received the highest ‘did not have a lot of information’ rating (24.7%). This concern has been highlighted in the European Parkinson's Disease Standards of Care, which states that most GPs are less familiar with the symptoms and necessary procedures of care for PD than a PD specialist [14,44,45]. Although perhaps understandable (GPs by necessity cannot specialize in all conditions under their care [46]), optimal management of PD symptoms requires detailed knowledge of the disease [47]. Many patients currently receive PD care from GPs, so these clinicians must either increase their understanding of the symptoms and appropriate care of PD patients or refer their patients to a specialist who can ensure adequate treatment and support. Regardless, GPs continue to play an important role in the care for PD patients, for example, by addressing comorbidity issues [48,49].

The Internet has become an important source of medical information [50–52], and the survey results reflect this as Internet searches were considered to be ‘very helpful’ by 51.8% of patients. Translation of health information Web sites into local languages could increase the accessibility and helpfulness further [52], which is particularly important in relation to Web sites providing information on medication options and clinical research, as these were the areas where the majority of patients (51.0%) indicated additional support would be beneficial. There is a need for an increased understanding of basic health information and services required to make appropriate health decisions amongst the general public, known as ‘eHealth literacy’ [53]. Initiatives to encourage this amongst PD patients could help them to obtain the most out of the online support available. The survey participants indicated that, although they may have access to one or all of the investigated services, each service ultimately ‘does not have enough time’ for them. Because the level of support provided by the Internet was considered to be high, it is feasible to consider introducing online information to patients at an early stage of their treatment to supplement the care provided by their clinician. However, as with all disease management support, evidence will be required to support clinical- and cost-effectiveness of such initiatives.

There are several limitations to this study, most notably its online nature. As highlighted in Part I [13], because the survey was only available online, participation required access to an Internet-connected computer. This potentially excludes a large number of the more elderly and disabled PD population, which may account for the relatively young survey population. However, online surveys may receive a greater number of responses than those sent through the post [54] and are convenient for PD patients to complete at home. A further limitation could be the potential overrepresentation of PD organizations due to the main method of promotion of the survey: translated Web site banners on PD organization Web sites. The participants could have an increased awareness of the EPDA and national PD organizations and therefore rate the accessibility and helpfulness of support from these services more highly. A further limitation is potential variability in the need for particular services due to the individual nature of the disease; a PD patient who does not have significant speech or swallowing problems would not require a speech therapist and could therefore be unaware of having access to this service.

Finally, as in the Part I manuscript, there is also a potential under- or overrepresentation of the participating countries; the level of response from each country is not necessarily indicative of the national population, and interpreting international differences should be performed with caution.

In conclusion, the results of the MfC Part II survey demonstrate that access to the complete multidisciplinary team members that are required to adequately support, treat, and care for PD patients is restricted across Europe. Despite the introduction of the EPDA Charter in 1997, patients are struggling to access allied health services such as OTs, podiatrists, and counselors. Furthermore, even when the services are accessible, they are not always considered to be particularly helpful. We hope that these results, in conjunction with the results from Part I of the survey, will raise awareness of the inequalities and shortfalls in the standards of care currently provided to PD patients and their families across Europe.

## Supporting Information

**Data S1.** Original questionnaire used for Move for Change Part II survey
