# Supplementary material for: Move for Change Part II: a European survey evaluating the impact of the EPDA Charter for people with Parkinson's disease
Source: Eur J Neurol. 2012 Oct 3;20(3):461–72. doi: 10.1111/j.1468-1331.2012.03876.x (PMC3593160; doi:10.1111/j.1468-1331.2012.03876.x)
Supplement: Supplementary file 1 [file ene0020-0461-SD1.docx]

**Online only: Original questionnaire used for Move for Change Part II survey**

| **1** | **Do you have Parkinson's disease?**  Please select one |  | Yes |
| --- | --- | --- | --- |
|  |  |  | No |
|  | Yes:  What year were you diagnosed with Parkinson’s? (yyyy) |  | |
|  |  |  | |
|  | How many years have you had Parkinson’s? |  | |
|  | No: |  |  |
|  | Are you: |  | Spouse |
|  |  |  | Partner or carer |
|  | How many years have you cared for someone with Parkinson’s? |  | |
| **2** | **Are you male or female?** |  | Male |
|  |  |  | Female |
|  |  |  | |
| **3** | **What is your age?**  Please select one |  | Under 30 |
|  |  |  | 30 - 39 |
|  |  |  | 40 - 49 |
|  |  |  | 50 - 59 |
|  |  |  | 60 - 69 |
|  |  |  | 70 - 79 |
|  |  |  | 80 and over |
| **4** | **In which country do you live?** |  | |

| **5** | **Which aspects of your life do you feel you need support with?**  Please tick all that apply | | | |
| --- | --- | --- | --- | --- |
|  |  | I do not need any support at this time | Finding opportunities for: | |
|  |  | Financial advice |  | Work |
|  |  | Emotional support |  | Activities to replace lost employment |
|  |  | Day-to-day living (mobility, household activities such as cooking, cleaning etc.) |  | Socializing with friends and family |
|  |  | Personal aspects (sex, sensuality and intimacy) |  | Meeting other people with Parkinson’s |
|  |  | Changes in relationships with family and friends |  | Participating in clinical trials |
|  |  | Understanding the disease symptoms |  | Participating in research studies |
|  |  | Learning about medication and treatment options that are available |  |  |
|  |  | Learning more about ongoing research |  |  |
|  |  | Going to work and continuing to work |  |  |
|  |  | Providing support for my family |  |  |
|  |  | Attending events related to Parkinson’s disease (e.g. educational courses and conferences) |  |  |
|  | Comments: | | | |

| **6** | **Have you had access to the following:**  Please select one | It does not apply | It is not available | It is available but I cannot afford it | It is available but I cannot get access | I do not know if it is available where I live | I have had access in the past but not now – I still require it | I have access when needed | I see them at least every  3 months | I see them at least every  6 months | I see them at least every  9 months | I see them at least every  12 months | I see them very rarely |
| --- | --- | --- | --- | --- | --- | --- | --- | --- | --- | --- | --- | --- | --- |
| a | Family doctor or general practitioner |  |  |  |  |  |  |  |  |  |  |  |  |
|  |  | Comments: | | | | | | | | | | | |
| b | Hospital doctor |  |  |  |  |  |  |  |  |  |  |  |  |
|  |  | Comments: | | | | | | | | | | | |
| c | Neurologist |  |  |  |  |  |  |  |  |  |  |  |  |
|  |  | Comments: | | | | | | | | | | | |
| d | Doctor with a special interest in Parkinson’s |  |  |  |  |  |  |  |  |  |  |  |  |
|  |  | Comments: | | | | | | | | | | | |
| e | Physician specializing in care of the elderly/geriatrician |  |  |  |  |  |  |  |  |  |  |  |  |
|  |  | Comments | | | | | | | | | | | |
| f | Parkinson’s disease nurse specialist |  |  |  |  |  |  |  |  |  |  |  |  |
|  |  | Comments: | | | | | | | | | | | |
| g | Physiotherapist |  |  |  |  |  |  |  |  |  |  |  |  |
|  |  | Comments: | | | | | | | | | | | |
| h | Occupational therapist |  |  |  |  |  |  |  |  |  |  |  |  |
|  |  | Comments: | | | | | | | | | | | |
| i | Speech and language therapist |  |  |  |  |  |  |  |  |  |  |  |  |
|  |  | Comments: | | | | | | | | | | | |
| j | Dietician |  |  |  |  |  |  |  |  |  |  |  |  |
|  |  | Comments: | | | | | | | | | | | |
| k | Podiatrist |  |  |  |  |  |  |  |  |  |  |  |  |
|  |  | Comments: | | | | | | | | | | | |
| l | Counseling |  |  |  |  |  |  |  |  |  |  |  |  |
|  |  | Comments: | | | | | | | | | | | |
| m | Psychologist |  |  |  |  |  |  |  |  |  |  |  |  |
|  |  | Comments: | | | | | | | | | | | |
| n | Social worker |  |  |  |  |  |  |  |  |  |  |  |  |
|  |  | Comments: | | | | | | | | | | | |
| o | Parkinson’s disease organization |  |  |  |  |  |  |  |  |  |  |  |  |
|  |  | Comments: | | | | | | | | | | | |
| p | Online support |  |  |  |  |  |  |  |  |  |  |  |  |
|  |  | Comments: | | | | | | | | | | | |
| q | Other support services you have received but not listed above  (e.g. t’ai chi, Nordic walking etc.) |  |  |  |  |  |  |  |  |  |  |  |  |
|  |  | Comments: | | | | | | | | | | | |

| **7** | **Which of the following helped you to get in touch with a support service?**  Please select one | It does not apply | But they were not very helpful | But they did not have much information | They were very helpful |
| --- | --- | --- | --- | --- | --- |
| a | Family doctor or general practitioner |  |  |  |  |
|  |  | Comments: | | | |
| b | Hospital doctor |  |  |  |  |
|  |  | Comments: | | | |
| c | Neurologist |  |  |  |  |
|  |  | Comments: | | | |
| d | Doctor with a special interest in Parkinson’s |  |  |  |  |
|  |  | Comments: | | | |
| e | Physician specializing in care of the elderly/geriatrician |  |  |  |  |
|  |  | Comments: | | | |
| f | Parkinson’s disease nurse specialist |  |  |  |  |
|  |  | Comments: | | | |
| g | Physiotherapist |  |  |  |  |
|  |  | Comments: | | | |
| h | Occupational therapist |  |  |  |  |
|  |  | Comments: | | | |
| i | Speech and language therapist |  |  |  |  |
|  |  | Comments: | | | |
| j | Dietician |  |  |  |  |
|  |  | Comments: | | | |
| k | Podiatrist |  |  |  |  |
|  |  | Comments: | | | |
| l | Counselor |  |  |  |  |
|  |  | Comments: | | | |
| m | Psychiatrist |  |  |  |  |
|  |  | Comments: | | | |
| n | Social worker |  |  |  |  |
|  |  | Comments: | | | |
| o | Parkinson’s disease organization |  |  |  |  |
|  |  | Comments: | | | |
| p | Online support |  |  |  |  |
|  |  | Comments: | | | |
| q | Friend |  |  |  |  |
|  |  | Comments: | | | |
| r | Family |  |  |  |  |
|  |  | Comments: | | | |
| s | I found information via Internet search engine  (e.g. Google) |  |  |  |  |
|  |  | Comments: | | | |
| t | Information received through the post |  |  |  |  |
|  |  | Comments: | | | |
| u | Information obtained whilst attending an event  (e.g. educational course, conference, awareness campaign) |  |  |  |  |
|  |  | Comments: | | | |
| v | other |  |  |  |  |
|  |  | Comments: | | | |

| **8** | **Do you feel the following support services have been helpful to you in managing your Parkinson’s?**  Please select one | It does not apply | Not very helpful | Very helpful | But there is not enough time |
| --- | --- | --- | --- | --- | --- |
| a | Family doctor or general practitioner |  |  |  |  |
|  |  | Comments: | | | |
| b | Hospital doctor |  |  |  |  |
|  |  | Comments: | | | |
| c | Neurologist |  |  |  |  |
|  |  | Comments: | | | |
| d | Doctor with a special interest in Parkinson’s |  |  |  |  |
|  |  | Comments: | | | |
| e | Physician specializing in care of the elderly/geriatrician |  |  |  |  |
|  |  | Comments: | | | |
| f | Parkinson’s disease nurse specialist |  |  |  |  |
|  |  | Comments: | | | |
| g | Physiotherapist |  |  |  |  |
|  |  | Comments: | | | |
| h | Occupational therapist |  |  |  |  |
|  |  | Comments: | | | |
| i | Speech and language therapist |  |  |  |  |
|  |  | Comments: | | | |
| j | Dietician |  |  |  |  |
|  |  | Comments: | | | |
| k | Podiatrist |  |  |  |  |
|  |  | Comments: | | | |
| l | Counselor |  |  |  |  |
|  |  | Comments: | | | |
| m | Social worker |  |  |  |  |
|  |  | Comments: | | | |
| n | Parkinson’s disease organization |  |  |  |  |
|  |  | Comments: | | | |
| o | Online support |  |  |  |  |
|  |  | Comments: | | | |
| p | Other |  |  |  |  |
|  |  | Comments: | | | |

| **9** | **How are the following support services funded?**  Please select one | It does not apply | By the government | By private insurance | I pay for this myself | By a Parkinson’s organization | By another organization |
| --- | --- | --- | --- | --- | --- | --- | --- |
| a | Family doctor or general practitioner |  |  |  |  |  |  |
|  |  | Comments: | | | | | |
| b | Hospital doctor |  |  |  |  |  |  |
|  |  | Comments: | | | | | |
| c | Neurologist |  |  |  |  |  |  |
|  |  | Comments: | | | | | |
| d | Doctor with a special interest in Parkinson’s |  |  |  |  |  |  |
|  |  | Comments: | | | | | |
| e | Physician specializing in care of the elderly/geriatrician |  |  |  |  |  |  |
|  |  | Comments | | | | | |
| f | Parkinson’s disease nurse specialist |  |  |  |  |  |  |
|  |  | Comments: | | | | | |
| g | Physiotherapist |  |  |  |  |  |  |
|  |  | Comments: | | | | | |
| h | Occupational therapist |  |  |  |  |  |  |
|  |  | Comments: | | | | | |
| i | Speech and language therapist |  |  |  |  |  |  |
|  |  | Comments: | | | | | |
| j | Dietician |  |  |  |  |  |  |
|  |  | Comments: | | | | | |
| k | Podiatrist |  |  |  |  |  |  |
|  |  | Comments: | | | | | |
| l | Counselor |  |  |  |  |  |  |
|  |  | Comments: | | | | | |
| m | Other |  |  |  |  |  |  |
|  |  | Comments: | | | | | |
